# Internal structure of cesium-bearing radioactive microparticles released from Fukushima nuclear power plant

**DOI:** 10.1038/srep20548

**Published:** 2016-02-03

**Authors:** Noriko Yamaguchi, Masanori Mitome, Akiyama-Hasegawa Kotone, Maki Asano, Kouji Adachi, Toshihiro Kogure

**Affiliations:** 1 National Institute for Agro-Environmental Sciences, Kannondai, Tsukuba 305-0864, Japan; 2 National Institute for Materials Science, Namiki, Tsukuba 305-0044, Japan; 3 Graduate School of Life and Environmental Sciences, University of Tsukuba, Tennodai, Tsukuba, 305-8572, Japan; 4 Meteorological Research Institute, Nagamine, Tsukuba, 305-0052, Japan; 5 Graduate School of Sciences, The University of Tokyo, Bunkyo, Tokyo 113-0033, Japan

## Abstract

Microparticles containing substantial amounts of radiocesium collected from the ground in Fukushima were investigated mainly by transmission electron microscopy (TEM) and X-ray microanalysis with scanning TEM (STEM). Particles of around 2 μm in diameter are basically silicate glass containing Fe and Zn as transition metals, Cs, Rb and K as alkali ions, and Sn as substantial elements. These elements are homogeneously distributed in the glass except Cs which has a concentration gradient, increasing from center to surface. Nano-sized crystallites such as copper- zinc- and molybdenum sulfide, and silver telluride were found inside the microparticles, which probably resulted from the segregation of the silicate and sulfide (telluride) during molten-stage. An alkali-depleted layer of ca. 0.2 μm thick exists at the outer side of the particle collected from cedar leaves 8 months after the nuclear accident, suggesting gradual leaching of radiocesium from the microparticles in the natural environment.

Although almost five years have passed since the accident of Fukushima Daiichi Nuclear Power Plant (FDNPP), radioactive contamination in the surrounding area is still a serious problem in Japan. The largest radionuclide deposition event occurred on March 15–16 and the second largest on 21–23, 2011. Wet deposition was a major source of radiocesium contamination of terrestrial environment^1^, while contribution of dry deposition was larger near the FDNPP^2^. In order to understand and predict the fate of radioactive materials contaminating the terrestrial environment, it is important to clarify the physicochemical properties of the deposited materials. From previous cases of radionuclide release, it is known that the chemical species of released radiocesium is monovalent cation (Cs^+^) which is soluble^3^. Deposition of radiocesium as insoluble particles has also been pointed out. Autoradiography analyses using imaging plate (IP) showed spots of particulate materials on plant tissues collected from Fukushima^4^,^5^,^6^. On the aerosol filter collected from March 14–15, 2011 in Tsukuba, 170 km south-southwest of FDNPP, Adachi *et al*.^7^ discovered spherical particulate radiocesium of 2.0–2.6 μm in diameter, with particles insoluble in water having a glass-like structure^8^. These microparticles contain several fission products of U-235 other than radiocesium, and Fe and Zn which are also used in nuclear reactors^8^. Hence, they were considered to be released directly from nuclear reactors.

Kaneyasu *et al*.^9^ suggested that vaporized radiocesium was transported with sulfate aerosol in the air, dissolved to cloud droplets and fell as rain. On the aerosol filter collected on March 20–21, 2011, rainy days in Tsukuba, the majority of radiocesium was in water-soluble form^7^. Such water-soluble radiocesium that reached the ground surface as a solute was fixed to soils, especially to clay minerals^10^. In the terrestrial environment, the majority of radiocesium is present in solid form regardless of the initial form of deposition. However, compared to clay minerals originally contaminated by soluble radiocesium in soil, the solid radiocesium, which was initially deposited as radioactive microparticles, had stronger radioactivity. Although the contribution or percentage of such radioactive microparticles in the contamination level of Fukushima has not been evaluated, its influence on human health may be serious in terms of its intense radioactivity. Moreover, the structural detail of the microparticles may give insights into the state of the broken reactor and fuel debris.

In the present study, we investigated radioactive microparticles, similar to those reported by Adachi *et al*.^7^, but collected from the ground, by observing their internal structure with transmission electron microscopic (TEM) techniques.

## Results

### Structure and composition of Cesium-bearing radioactive microparticles

Cesium-bearing radioactive microparticles that had been deposited on non-woven fabric cloth (NWC-1) and on a needle of Japanese cedar (*Cryptomeria japonica*) (CB-8) were investigated. They were in the field for five and eight months, respectively, until sampling. Scanning electron microscope (SEM) images of NWC-1 of the whole microparticle before preparing thin sections for TEM analyses; and elemental composition of the whole particle determined by synchrotron radiation microbeam X-ray fluorescence (SXRF) are shown in Supplementary Figs S1 and S2 online, respectively. The activities of ^137^Cs for the NWC-1 and CB-8 were 5.04 ± 0.472 and 3.14 ± 0.178 Bq, respectively.

**Figure 1:**
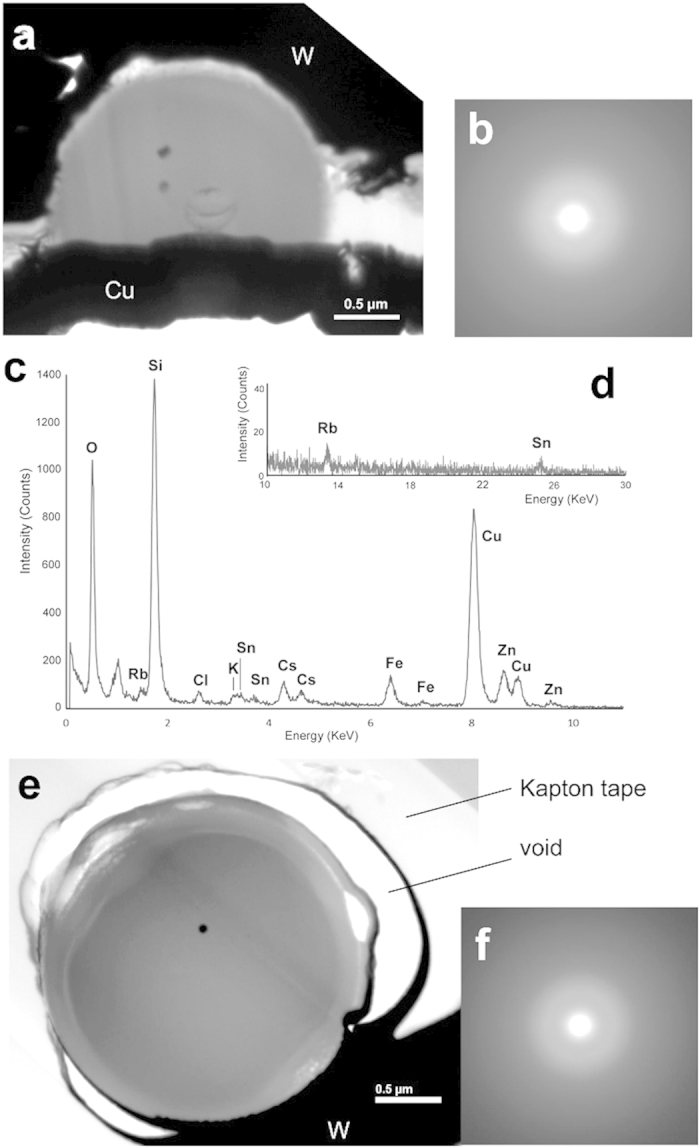
(**a**) Bright-field (BF) image of NWC-1. The opaque material outside the microparticle is tungsten (W) and copper (Cu) deposited in the FIB process. (**b**) Electron diffraction (ED) pattern from the particle. (**c,d**) EDS spectrum acquired from almost the whole area of the particle for the energy range of (**c**) 0–11 keV and (**d**) 10–30 keV. (**e**) BF image of CB-8. The opaque material outside the sphere is W and the thin transparent material is Kapton tape. (**f**) ED pattern from the particle.

Bright-field (BF) images and selected-area electron diffraction (SAED) patterns recorded in TEM from the whole area of NWC-1 and CB-8 are shown in Fig. 1, along with the energy-dispersive X-ray spectrum (EDS) acquired in TEM from NWC-1. Preparation of thin specimens from NWC-1 microparticle by focused-ion-beam (FIB) process was successful only for the upper half of the sphere. The BF contrast of NWC-1 (Fig. 1a) was almost uniform except for two dark nanoparticles inside the sphere. These nanoparticles will be described in a later subsection. The SAED pattern from the whole particle consists of only a halo (Fig. 1b) and the EDS from the whole particle (Fig. 1c,d) mainly consist of peaks of Si and O (Cu is from the supporting mesh), indicating that the particle was basically silicate glass. Additionally, Cl, K, Cs, Fe, Zn, Rb, and Sn were definitely identified. The presence of Na was not confirmed since its peak overlapped with the L-peak of Zn. These elements were previously reported^7^,^8^ except for K. EDS Semi-quantitative analysis for almost the entire area of the TEM specimen, without considering the absorption effect estimated the glass composition (wt.%) to be SiO_2_; 69.3, K_2_O; 1.9, Fe_2_O_3_; 8.6, ZnO; 11.0, Rb_2_O; 1.3, SnO_2_; 1.4, Cs_2_O; 3.4 with a small amount of Cl (1.4 wt.%). The valence states of cations were assumed to be in line with results in an X-ray absorption spectroscopic study^8^. Rubidium, Cs and Sn are the fission products of U-235. Tin is also used for fuel cladding^11^. Iron is used as steel for reactor pressure vessels, and Si and O are main components of concrete at the bottom of the containment vessel, where the melt-down fuel or core debris is thought to exist. Zinc had been added to the primary cooling water^12^. Potassium and Cl may originate from concrete and/or seawater, which was used to cool down the reactor.

The BF TEM image of CB-8 showed a two-layer structure having an inner core with darker contrast, an outer crust with a lighter one, and a small dark nanoparticle near the center (Fig. 1e). The thickness of the crust was around 0.2 μm. SAED pattern indicated that both are amorphous (Fig. 1f). Locally, a bubble-like structure was observed at a certain radius in the crust, or the outside part was peeled off at the radius (Fig. 1e). The chemical composition of the entire area of the specimen was SiO_2_; 73.3, K_2_O; 1.4, Fe_2_O_3_; 7.2, ZnO; 11.4, Rb_2_O; 1.2, SnO_2_; 1.5, Cs_2_O; 3.3 and Cl; 0.7, which is similar to that of NWC-1.

### Distributions of elements in microparticles

**Figure 2:**
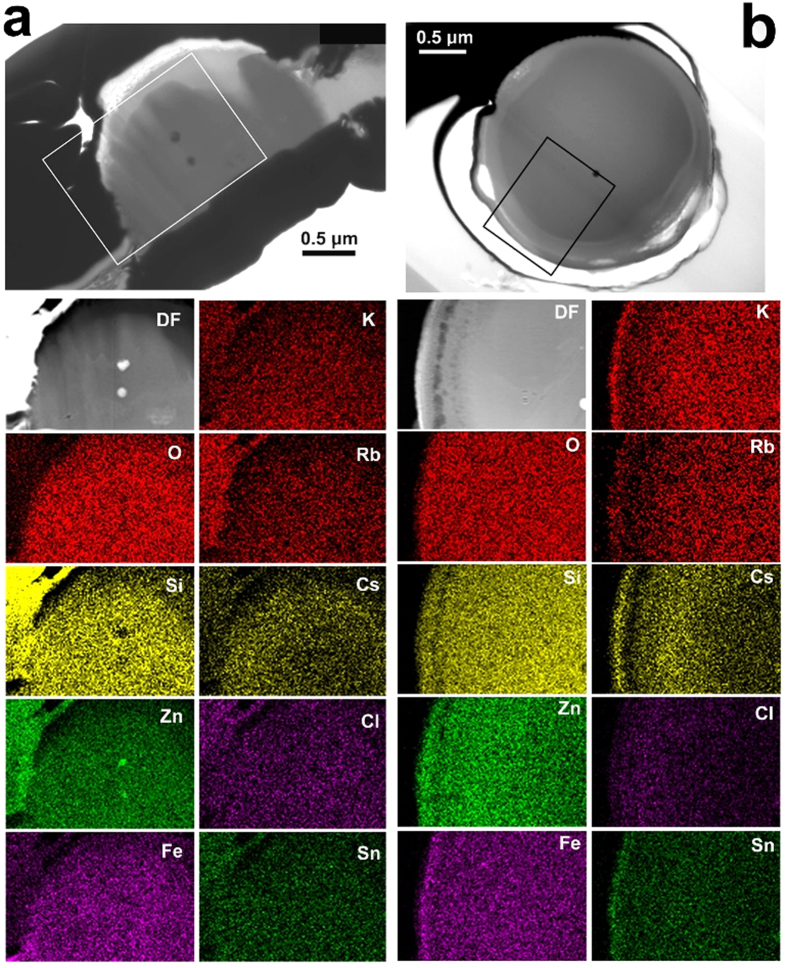
(**a**) Element maps of NWC-1 acquired by STEM-EDS with a STEM Dark-field (DF) image at the same area, and (top) TEM Bright-field image in which the rectangle indicates the area analyzed by STEM-EDS. The thin area around the top of the particle which was not observed in Fig. 1a was formed by a further thinning process by FIB. (**b**) Element maps and images of CB-8 with the same framing as in (**a**).

Elemental maps from NWC-1 using STEM-EDS are shown in Fig. 2a. Brighter color indicates higher concentration of the elements. All elements were almost uniformly distributed in the particle with Cs the only exception. Cesium concentration was lower near the center than the outside of the particle. The ratio of the amounts between the center and outside is around two. It is possible that vapor-phase cesium in the reactor was absorbed to the molten silicate microparticles, from their surface. The low diffusion velocity of cesium in the glass may have left the concentration gradient. On the contrary, elemental mapping for CB-8 (Fig. 2b) indicates that K, Rb and Cs were distinctively depleted in the crust but concentrated in the vicinity of the surface. Although the reason for the high concentration at the surface is not certain, it might be an artifact by diffusion of alkali cations caused by electron-beam radiation in the analysis. Chlorine was concentrated in the crust.

### Crystalline nanoparticles in the glass

**Figure 3:**
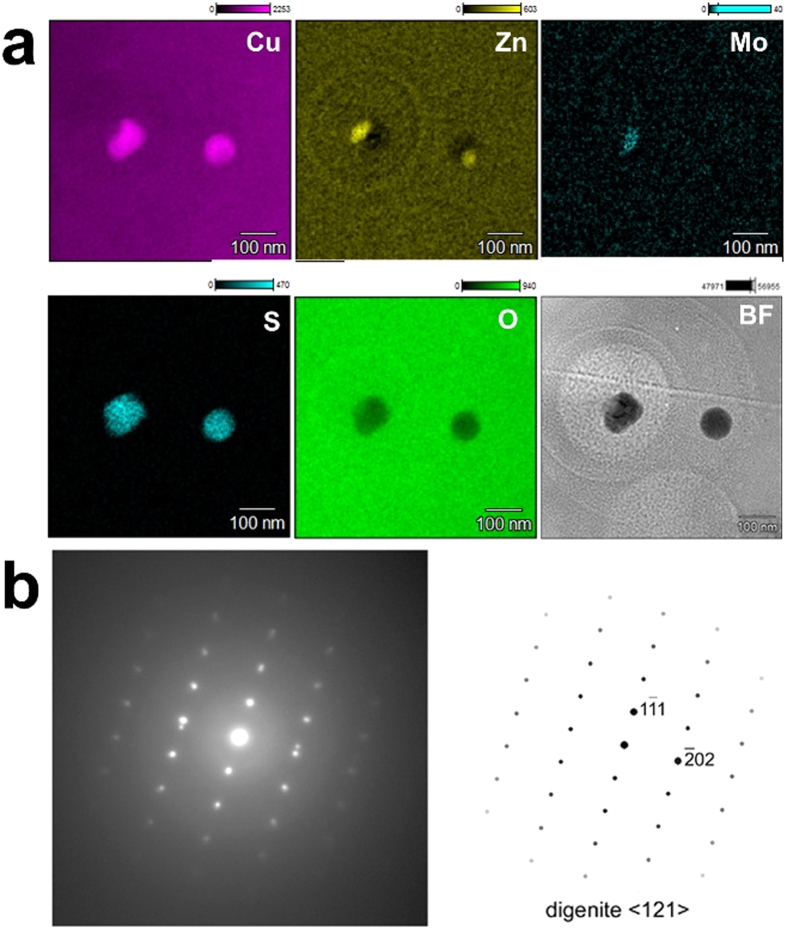
(**a**) Element maps and STEM bright-field (BF) image of the two nanoparticles inside NWC-1. The circular contrasts outside the nanoparticles in the BF image are radiation damage in glass formed by the electron probe for chemical analyses. (**b**) (Left) ED pattern from the left nanoparticle. (Right) Drawing of ED pattern for the high-temperature polymorph of digenite (Cu_2−x_S) along <121>.

Two nanoparticles with dark contrast were observed in the TEM image of NWC-1 (Fig. 1a). Although EDS spectrum only from the particles cannot be obtained since they are still buried in the glass in spite of the thinning by FIB, S was distinctively identified from the particles (Fig. 3a). Elemental mapping by STEM-EDS indicated enrichment of Cu, Zn and Mo at the nanoparticles along with S, indicating that they are sulfide. The possibility of sulfate was excluded because oxygen is deficient in the nanoparticles in the oxygen map (Fig. 3a). Moreover, the locations of Cu, Zn and Mo do not overlap within the nanoparticles, suggesting that these metal elements form different sulfide phases in the nanoparticle (Fig. 3a). SAED from the left particle showed a single-crystal diffraction pattern (Fig. 3b), which can be explained by the crystallographic parameters for a high-temperature polymorph of digenite (Cu_2−x_S). The origin of Cu and S may be minor elements in the concrete. On the other hand, Ag and Te were distinctively detected by EDS in TEM from the nanoparticle in CB-8 (Supplementary Fig. S3 online), indicating the particle to be silver telluride. Both elements can be fission products of U. Diffraction spots were observed in the SAED pattern from the particle, indicating that it is crystalline but the phase could not be identified. These sulfide and telluride were probably segregated and crystallized in the microparticles at the molten state.

## Discussion

Our most significant finding is that the matrix of the Cs-bearing microparticles is silicate glass, based on the TEM-EDS analysis with FIB sample preparation. Previous studies suggested that Fe, Mo, Sn and Zn in the Cs-bearing microparticles had a similar X-ray absorption near-edge structure to those composed of glass^8^, however the presence of Si in the microparticles has not been verified^7^,^8^. It is probable that the high-temperature melt-down fuel from the reactor came into contact with and melted the concrete, and then splashed microparticles of silicate melt, which were solidified by cooling to form silicate glass in the atmosphere. However, there are several questions with respect to the selection of the constituting elements in the glass from various ones in the reactor. For instance, Ca which is one of the major elements in concrete, was almost absent in the microparticles of NWC-1. Since TEM observed only a small portion of the microparticles, by making them thin using FIB, there may have been other elements in the microparticles, for instance, as a form of chalcogenide nanoparticle.

The next important finding is the alkali-depleted crust in CB-8 microparticle. This is probably the result of elution of alkali ions by contact with acidic solution in the field, commonly observed in silicate glass^13^. On the other hand, such alkali-depleted crust was not observed in NWC-1. This may be attributed to the different environments of the two microparticles after release from the nuclear plant. NWC-1 was on non-woven cloth and CB-8 on a cedar leaf before it was collected. It is well-known that silicate glass elutes alkali components from their surface by ion-exchange with proton or hydronium ions to form an alkali-leaching layer on the surface if pH of reacting solution is low, whereas the silicate framework of the glass itself is dissolved with high-pH solution^13^,^14^. Coniferous forest canopy induces acidic condition due to ammonia uptake, nitrification and leaching of plant-derived acid^15^. It was likely that CB-8 deposited on the cedar leaf had been in acidic conditions, which derives alkali depletion in the crust, for eight months. The finding of the alkali-depleting crust on the surface of the Cs-bearing radioactive microparticle indicates that radiocesium in the particles can be released by “weathering” of the glass in natural environments, and considering its small size, duration for the total release of the radioactive cesium from the particles is probably not long, from several years to a few decades, though it will strongly depends on the environment.

In order to investigate the dissolution rate and detailed Cs-leaching properties of the Cs-bearing radioactive microparticles, a leaching experiment should be conducted as a function of temperature and pH. However, collecting and isolating the Cs-bearing microparticles is time-consuming and it is difficult to obtain a large enough number of Cs-bearing microparticles to investigate dissolution properties. Alternatively, synthesized silicate glass with the same composition as the microparticles presented in this study may help to obtain information on the fate of Cs-bearing radioactive microparticles. However, in our preliminary experiment, we were unable to make uniform glass with the same composition at present. This is probably due to the liquid immiscibility at the composition. A solution to this problem is now being considered.

There are mainly two types of solid-phase radiocesium in the terrestrial environment affected by FDNPP accident; that fixed to clay minerals in the soil via wet deposition and that contained in the microparticles of silicate glass flown directly from the nuclear reactors. The radioactivity of the former is rather in low-density but distributed widely, therefore it is a major source of external radiation from ground. Part of the radiocesium adsorbed on the soil clay is desorbed and transferred to crops thereby causing internal radiation. On the other hand, contribution of the microparticles to the air radiation is most likely not significant, but their radiation density is very high, which is particularly problematic for organisms including humans if the microparticles are inhaled or ingested. The plant availability of radiocesium in the microparticles should depend on its solubility. Consequently, further research on this material should be carried out as soon as possible.

## Methods

### Samples

Radioactive microparticles attached on non-woven fabric cloth and needles of Japanese cedar, collected from Fukushima, were identified by autoradiography followed by point-by-point analyses with scanning electron microscope (SEM; Hitachi high-Technologies SU3500) equipped with an energy dispersed X-ray spectrometer (EDS; Horiba X-max 50 mm). The identified particles were further determined by SXRF with excitation X-ray energy of 37.5 keV to ensure the detection of Cs-K line from the particle. The activities of ^137^Cs was determined by a Ge detector (GCW2523S Canberra, USA). Detailed methods to identify the Cs-bearing microparticles are described in the supplementary information.

### TEM Analyses

Cross-sectional thin TEM specimens were prepared from radioactive microparticles using a focused ion beam (FIB) instrument with micro-sampling system (Hitachi FB-2100) as described in the supplementary information. Then specimens were initially examined using a TEM (JEOL JEM-2010UHR) operated at 200 kV with an EDS analyzer system (JEOL JED-2200). Elemental mapping in the microparticles and quantitative analyses were performed using a JEOL JEM-3100FEF operated at 300 kV in the STEM mode, with an EDS analyzer system (Thermo Fisher Scientific NORAN System SIX). Finally, elemental maps for nanoparticulates inside the microparticles were acquired using a JEOL JEM-2800 operated at 200 kV with double wide-area (0.95 sr.) silicon drift detectors (SDD) for EDS analyses.

## Additional Information

**How to cite this article**: Yamaguchi, N. *et al*. Internal structure of cesium-bearing radioactive microparticles released from Fukushima nuclear power plant. *Sci. Rep.* **6**, 20548; doi: 10.1038/srep20548 (2016).

## Supplementary Material

Supplementary Information
