# Supplementary material for: Internal structure of cesium-bearing radioactive microparticles released from Fukushima nuclear power plant
Source: Sci Rep. 2016 Feb 3;6:20548. doi: 10.1038/srep20548 (PMC4738348; doi:10.1038/srep20548)
Supplement: Supplementary Information [file srep20548-s1.pdf]

Supplementary information for

Internal structure of cesium-bearing radioactive microparticles released from Fukushima nuclear plant

Noriko Yamaguchi,[1*] Masanori Mitome[2], Kotone Akiyama-Hasegawa[2], Maki Asano[1, 3], Kouji Adachi[4] and Toshihiro Kogure[5*]

[1]National Institute for Agro-Environmental Sciences,

[2]National Institute for Materials Science,

[3]Graduate School of Life and Environmental Sciences, University of Tsukuba

[4]Meteorological Research Institute

[5]Graduate School of Sciences, The University of Tokyo

*corresponding authors:

Noriko Yamaguchi, 3-1-3 Kannondai, Tsukuba 305-0864, Japan

Tel: +81-29-838-8315, nyamag@affrc.go.jp

Toshihiro Kogure, Bunkyo, Tokyo 113-0033, Japan.

Tel: +81-3-5841-4019, kogure@eps.s.u-tokyo.ac.jp

Details of the Experiment

Samples

Cesium-bearing radioactive microparticles were taken from the deposited particles on non-woven fabric cloth (NWC-1) and on a needle of Japanese cedar (Cryptomeria japonica) (CB-8). The non-woven fabric cloth was used as ground-cover mulcting material for leafy vegetable cultivation in Nihonmatsu, Fukushima, 50 km west from the nuclear plant. The cloth was left outside until August 2011 and then in roofed storehouse. Branches of Japanese cedar tree were taken from Ohtsubeyama Mountain in Kawauchi, Fukushima, 20 km south-west from the nuclear plant in November, 2011 and stored inside after air-drying. The non-woven fabric and needles of the Japanese cedar came into contact with an imaging plate for 10 min (BAS-2500, Fujifilm). The high radiation spot on the samples were identified based on the exposed area of the screen. The area around the high radioactivity spot was clipped in 5 mm square then the clipped piece was cut into 15~20 fragments. Each fragment came into contacted with an imaging plate again for 5 min and the fragment with high radioactivity was selected. The selected fragment was placed on Kapton tape. The segment with high radioactivity was isolated as far as possible by removing low-radioactivity residues by repeatedly paste and peel another Kapton tape and the radioactive segment was selected based on γ-ray counting by a Ge detector (GCW2523S Canberra, USA). The Kapton tape with isolated radioactive segment was placed on an imaging plate for overnight to ensure radioactive particle other than the isolated particle was not exist on the tape. The activities of ^{137}Cs of the isolated radioactive particles were determined by a Ge detector.

Cesium-bearing particles were identified by a scanning electron microscope (SEM; Hitachi high-Technologies SU3500) equipped with an energy dispersed X-ray spectrometer (EDS; Horiba ltd. X-max 50 mm). The identified particles were further determined by synchrotron radiation microbeam X-ray fluorescence (SXRF) at an undulator beamline 37XU of SPring-8. An incident X-ray beam of 37.5 keV was focused to the spot size of 1.2 μm in the vertical direction and 1.0 μm in the horizontal direction. The fluorescent X-rays, Cs Kα line (30.97 keV), Sn Kα line (25.19 keV), Mo, Kα line (17.44 keV), Rb Kα line (13.37 keV), Zn Kα line (8.62 keV) and Fe Kα line (6.40 keV) were detected using a Si(Li) solid-state detector. The scan step size and the spectral acquisition time for each spot were 1 μm and 0.5 s, respectively. The SXRF maps ensured that there were no Cs-bearing particles around the target particle. On the spot that localizing Cs, SXRF spectra were analyzed for 600 s and the result are shown in Fig. S2.

Preparation of Cross-sectional thin TEM specimens

Cross-sectional thin TEM specimens were prepared from the radioactive microparticles using a focused ion beam (FIB) instrument with micro-sampling system (Hitachi FB-2100). The microparticles on double-stick tape were previously coated with carbon to prevent charge accumulation during the FIB process. In the FIB procedure, tungsten was deposited on the target area of the microparticles. Then trenches were formed around the target area with high energy (30 keV) Ga ion beam, and a fragment of thin sections was transferred onto a crescent-shape cupper grid using the micro-sampling system. The fragment was thinned to around 200 nm with a low energy Ga ion beam of 10 keV so as to minimize the surface damage caused by the bombardment of Ga ion.

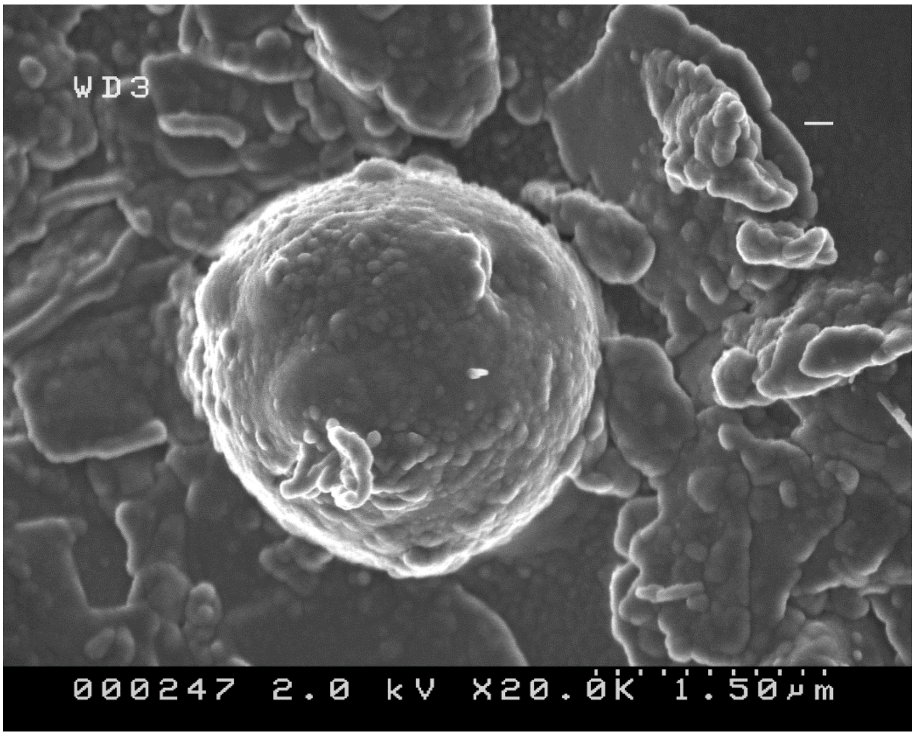

Figure S1 SEM (secondary electron) images of Cs-bearing microparticle deposited on non-woven fabric cloth collected from Nihonmatsu, Fukushima in 2011 (NWC-1). The image was taken using a Hitachi S-4500 SEM operated at 2.0 kV.

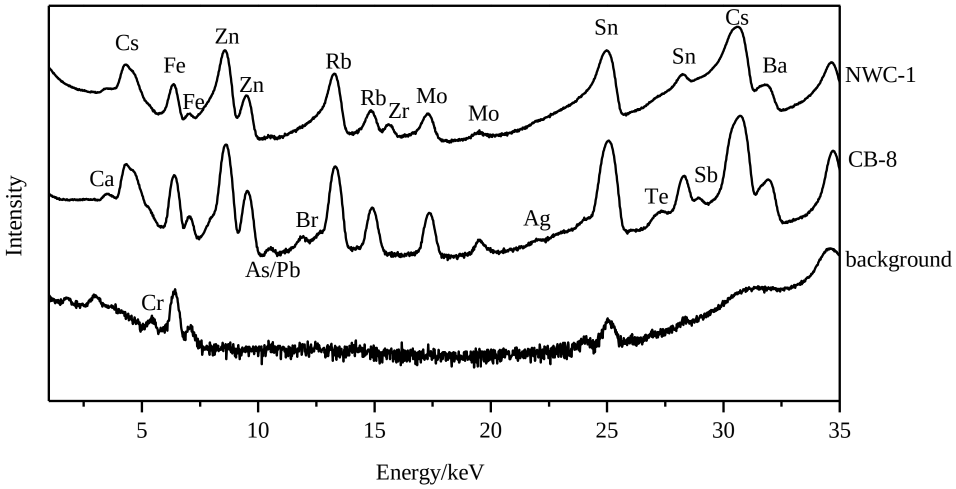

Figure S2. Synchrotron radiation microbeam X-ray fluorescence spectra. The intensity of each spectrum is plotted on a logarithmic scale and the y-axis has an offset for clarify. Besides the elements detected by TEM-EDS analyses, barium and zirconium were also detected but its contribution to the entire elemental composition was small.

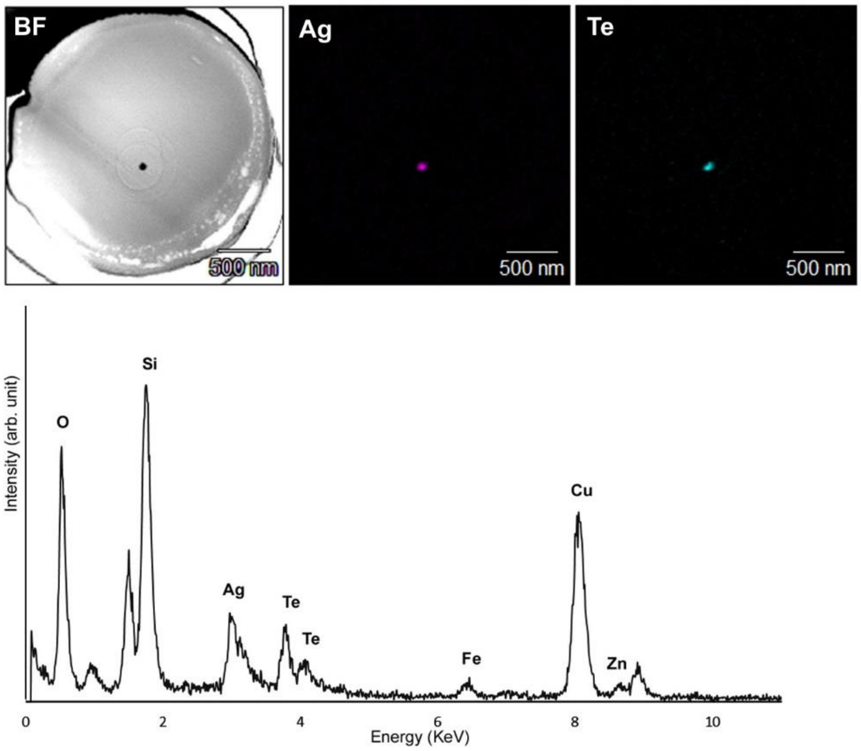

Figure S3. (Top) STEM bright-field (BF) image and element maps of Ag and Te, acquired from CB-8. (Bottom) EDS spectrum acquired with the electron probe placed on the opaque nanoparticle.
